# Effect of Moringa oleifera and ivermectin nanoparticles on the immunopathological response during experimental trichinosis in mice

**DOI:** 10.1186/s13099-025-00764-7

**Published:** 2025-11-14

**Authors:** Magda Said Ahmed Abdeltawab, Alshaimaa M. R. Hamed, Shimaa Saad El-Din, Engy Medhat, Mai Samir, Amal M. Mahfoz, Abdel Wahab M. Mahmoud, Basma Emad Aboulhoda, Hend Ahmed Abdallah, Hanaa S. Sallam, Mona Said El-Sherbini

**Affiliations:** 1 Department of Medical Parasitology, Faculty of Medicine, Cairo University, Cairo, Egypt; 2 Department of Medical Biochemistry and Molecular Biology, Faculty of Medicine, Cairo University, Cairo, Egypt; 3 Department of Medical Biochemistry and Molecular Biology, Faculty of Medicine, Badya University, Giza, Egypt; 4 Egyptian Drug Authority (EDA), Giza, Egypt; 5 Department of Plant Physiology, Faculty of Agriculture, Cairo University, Giza, Egypt; 6 Department of Anatomy and Embryology, Faculty of Medicine, Cairo University, Cairo, Egypt; 7 Department of Human Physiology, Faculty of Medicine, Suez Canal University, Ismailia, Egypt; 8 Department of Population Health and Health Disparities, Division of Global Partnerships, School of Public and Population Health, University of Texas Medical Branch at Galveston, Galveston, TX USA

**Keywords:** Trichinosis, Nanoparticles, Moringa oleifera, Macrophage polarization.

## Abstract

*Trichinella spiralis* (*T. spiralis*) infection dynamically modulates macrophage polarization. It promotes M1 macrophage polarization, enhancing the pro-inflammatory pathways. This study investigates how ivermectin nanoparticles (IVM-NP) and *Moringa oleifera* leaf extract (MOL-NP) regulate these pathways to improve the pathophysiological outcomes of trichinosis. Thirty Swiss albino mice were infected with *T. spiralis* and divided equally into five groups of six mice each: healthy controls, infected untreated, IVM-NP-treated, MOL-NP-treated, and combined IVM-NP and MOL-NP-treated. IVM-NP were administered as a single oral dose of 200 µg/kg at the beginning of the experiment. MOL-NP were delivered orally at a dose of 400 mg/kg/day for 5 consecutive days starting from experiment initiation. Parasitological examination to detect the parasitic burden in addition to histopathological, immunohistochemical and quantitative histomorphometric assessment of intestinal tissue for nuclear factor kappa B (NF-κB) and inducible nitric oxide synthase (iNOS) were done. Furthermore, RT-PCR was performed to evaluate the relative gene expression of Arginase-1, TNF-α, and IL-10. Treatment with nanoparticle formulations of IVM and MOL modulated macrophage-related immune responses by reducing the pro-inflammatory markers iNOS, TNF-α and NF-κB, while increasing the relative gene expression of the anti-inflammatory cytokine IL-10. Combination therapy exhibited superior efficacy in decreasing parasite burden and mitigating intestinal pathology compared to monotherapy.

## Introduction

The immune response is composed of an orchestra of fine-tuned interlacing pathways that exert varying defense strategies against various microbial threats. Macrophage polarization represents a vivid example of the delicate balance of immunological mechanisms. Macrophages are generally categorized into classically activated macrophages (M1) and alternatively activated macrophages (M2). These 2 spectra of macrophage phenotype represent the traditional poles of the immune response, the killing response against various threats versus the healing and regenerative potential after the resolution of combat [1]. M1 macrophages are stimulated by several pro-inflammatory mediators and augment the T helper (Th)1 immune response by producing pro-inflammatory chemokines and cytokines such as TNF-α and interleukins 1,6,12,18, and 23. In parallel, M2 macrophages enhance the Th2 immunity arm and are particularly important in parasitic infections. IL-10 serves both as an inducer and effector of alternatively activated macrophages [2].

A key marker of M2 macrophages, arginase-1, converts L-arginine into L-ornithine and urea. Induction or activation of this enzyme depletes L-arginine, limiting its use by inducible nitric oxide synthase (iNOS), which in turn is a marker for M1 macrophages. Inducible NOS converts L-arginine into nitric oxide (NO) and L-citrulline. NO is an important effector molecule in the process of phagocytosis and antimicrobial immune response [3]. Macrophage polarization is a dynamic and flexible process that adapts to the status of the immune-pathological response. An important regulator of macrophage phenotype switching is the nuclear factor kappa beta (NF-κB) pathway. During the early inflammatory phase of tumor formation, macrophages exhibit a high activity of NF-κB and M1 activity profile. Later in disease progression, an immunosuppressive state is established, which is characterized by an M2 phenotype and low NF-κB [4].

*Trichinella spiralis* infection has been employed as a rich and comprehensive model to study both local tissue-specific and systemic immune responses. Several signaling pathways synergize and interlace to shape the host reaction against this unique nematode. M1 polarization is an important feature of intestinal trichinellosis, where M1 macrophages play an important role in adult worm expulsion [5]. The intestinal phase of *T. spiralis* infection starts after the release of L1 larvae from the digested collagen capsules upon ingestion of infected meat. The freed larvae invade the small intestinal epithelium to mature into adult worms, which copulate at around day 7 post-infection (PI) to generate a new progeny of larvae, that will then migrate to finally encyst in skeletal muscles [6].

Herbal agents are successful synergistic and sometimes alternative remedies to conventional anti-parasitic agents [7].

*Moringa oleifera*, a member of the Moringaceae family, has been traditionally used as a medicinal plant due to its multiple health benefits, which include antioxidant, anti-hyperglycemic, anti-hyperlipidemic, and antihypertensive activities [8].

In addition to the rediscovery of herbal agents as potent anti-parasitic effectors, scientists sought to enhance the effect of conventional anti-parasitic drugs through the addition of nanoparticles. The integration of nanomedicine and anti-parasitic agents aims to overcome drug limitations such as low solubility and bioavailability, as well as high dosing and elimination [9]. Ivermectin (IVM), also termed the ‘wonder drug’, was shown to be effective against a variety of parasitic agents [10]. Nano-preparations of IVM include the employment of liposomes, solid lipid nanoparticles and nano-suspensions as drug delivery systems [11–13].

This study aims to investigate the potential impact of *Moringa oleifera* and IVM nanoparticles on immune signaling pathways, particularly those involved in macrophage polarization which are involved in the immune response against intestinal trichinosis.

## Materials and methods

### Nanoparticle preparation

Nanoparticles from a single production batch were used for all experiments to ensure consistent treatment conditions.

#### Ivermectin nanoparticles synthesis

Chitosan (MW 71.3 kDa, degree of deacetylation (89%) was purchased from Aldrich (Germany) while sodium alginate (77%) was purchased from Sigma Chemical Co. (St. Louis, USA). All other reagents were purchased from Aldrich (Germany).

Nanoparticles of IVM were prepared by a Top-Down molecular chemical approach, where chitosan and sodium alginate as carriers of Ivermectin were mixed 2:1 (*v: v*) at molar ratio, then 1.5 ml of methacrylic acid aqueous solution (0.7%) was added and left for 9 h continuously under magnetic stirring at 27 °C with drops of dimethyl sulfoxide. Ivermectin (2 gm powder dissolved in mixture deionized water and ethanol 95%) was added gradually to the previous mixture during stirring. Then, ethylhexanoate, polyethylene glycol (PEG) and polyoxyethylene polymer were incorporated into the formulation at 0.6% (v/v) each. After that, the mixture was incubated at 33 °C for 72 h continuously, followed by an 18-hour intermittent exposure to 1.5 pound per square inch (psi) pressure. Afterward, the mixture was centrifuged at 1500 rounds per minute (rpm) for 45 min, with further cooling in an ice bath. Then, the resulting precipitate was filtered and rigorously washed with deionized water to remove residual impurities [14, 15].

The prepared nanoparticles exhibited irregular shapes with an average size range of 32 to 39 nm with crystalline structure suspension and high purity (98.5%). Particles morphology and size were assessed using a JEOL 1010 transmission electron microscope (TEM), (JEOL, Japan) operating at 80 kV. For TEM imaging, a droplet of the nanoparticle suspension was deposited onto a carbon-coated copper grid and air-dried at room temperature. Nanoparticle dimensions were determined using Image-Pro Plus 4.5 software. The size values represent the mean of three independent measurements.

#### Synthesis of Moringa oleifera leaves nanoparticles

According to Marslin et al. [16] method with modification, *Moringa oleifera* plant leaves were air dried then 500 mg of dried leaves were grinded into fine powder then added to 50 mL of methanol and hexane 75% (1:1) for 48 h under 25 °C afterward, this solution was blundered for 5 min and filtered, subsequently 15 ml of the extract solution was added into warm water (35 °C) under ultrasonic conditions (power of 750 W and a frequency of 20 kHz) for 8 min. Following sonication, the mixture was stirred at 550 rpm for 30 min under room temperature, then centrifuged at 1200 rpm for 30 min. After that, cooling of the mixture was performed in an ice bath and then freeze-dried [17].

The resulting nanoparticles displayed polydisperse shapes with a size range of 15–21 nm, crystalline properties, and 98.5% purity. Characterization was performed as formerly described using TEM (JEOL 1010, 80 kV).

### Experimental infection

This study was conducted in compliance with the ethical guidelines for animal research and was approved by the Institutional Animal Care and Use Committee (IACUC) under protocol number CU III-F-6124 (3/2025). The experiment was done according to the National Research Council Guide for the Care and Use of Laboratory Animals and its related guidelines.

Infective L1 *T. spiralis* larvae were obtained from donor mice from a murine laboratory-maintained *T. spiralis* life cycle. After euthanasia, skeletal muscles from infected mice were collected and digested in 1% pepsin and 1% HCl solution at 37 °C for 2 h. During the digestion process, periodic agitation using an electric stirrer was performed to enhance larval liberation. The digested muscles were initially filtered through a 50-mesh sieve to eliminate undigested tissue fragments. Larvae were then collected using a finer 200-mesh sieve, subjected to two washing steps, and finally resuspended in 150 mL of tap water within a conical flask for subsequent processing. The larval suspension was allowed to sediment, after which the supernatant was discarded. Larvae were then collected and counted using a dissecting microscope to precisely calibrate the individual infective dose for each mouse [18–20].

The infective dose of L1 larvae was weight-adjusted, with each mouse receiving 10 larvae per gram body weight, making the dose 250 ± 50 larvae per mouse. Infection was performed by oral inoculation. For the study experiment, 30 laboratory-bred, pathogen-free Swiss albino mice (4–6 weeks old) weighing between 25 and 35 g were kept on a standard diet and under a temperature of 24 C°. They were divided equally into five groups:

Group I (Negative control): Healthy non-infected mice.

Group II (Pathological control): Infected non-treated group.

Group III (IVM-NP): Infected mice treated with IVM nanoparticles (IVM-NP).

Group IV (MOL-NP): Infected mice treated with *Moringa oleifera* leaf extract (MOL).

Group V (IVM-MOL): Infected mice treated with both IVM-NP and MOL-NP.

### Drug administration

Ivermectin nanoparticles (Iverzine 6 mg tablets, Uni Pharma, Egypt) and *Moringa oleifera* leaf extract were dissolved in saline. Dosages were determined through human-to-animal dose extrapolation according to established protocols [20]. Both drugs were given at the beginning of the experiment. IVM-NP were administered as a single oral dose of 200 µg/kg at the beginning of the experiment, while MOL-NP were delivered orally at a dose of 400 mg/Kg/day for 5 consecutive days. On day 7 post-infection (PI), animals were euthanized via cervical dislocation following proper anesthesia induction.

### Parasitological examination

On day 7 PI, intestinal worm burden was assessed by counting the number of adult worms per milliliter of intestinal fluid. The small intestines were longitudinally incised and thoroughly washed with saline and cut into small pieces. Tissue segments were incubated in phosphate-buffered saline (PBS, pH 7.4) at 37 °C for 2 h. Liberated adult worms were then collected and counted under a dissecting microscope [21].

### Histopathological examination

Intestinal tissue samples (1 cm from the small intestine at the junction of the proximal 1/3 and distal 2/3) [22] were harvested from euthanized mice [23, 24]. These tissues were fixed in 10% formalin, dehydrated, cleared in xylene and embedded in paraffin blocks. Serial Sect. (5 μm thickness) were prepared and stained with hematoxylin and eosin (H&E) for microscopic evaluation using a Leica Qwin light microscope to assess infection-induced histopathological alterations [25].

###  Immunohistochemical examination and histomorphometric assessment

The protein expression of iNOS and NF-κB was evaluated by immunohistochemistry [26]. Deparaffinized intestinal sections were placed on positively charged slides and antigen retrieval in citrate buffer (95 °C, 20 min) was performed after rehydration through graded alcohols and PBS rinses. Hydrogen peroxide was introduced for quenching of the endogenous peroxidase activity. Sections were incubated overnight at 4 °C with primary antibodies: rabbit anti-iNOS (Thermo Fisher MA5-16422, 1:100) or mouse anti-NF-κB (Thermo Fisher 33–9900, 1:100). Detection employed diaminobenzidine (DAB) chromogen with Mayer’s hematoxylin counterstain. The exact procedures were performed for the negative controls; however, the primary antibodies were not used.

Immunohistochemistry quantification was performed by an investigator blinded to treatment groups. The image J analysis system software (Image Pro Plus 6.0, Media Cybernetics, USA) was used to conduct quantitative histomorphometric analysis which evaluated the area percentage of iNOS immune-expression and the count of NF-κB immune-reactive cells in 6 non-overlapping microscopic fields at 400x magnification.

### Molecular assay

Small intestinal tissue samples were mechanically homogenized in lysis buffer and processed for RNA extraction using the GeneJET RNA Purification Kit (Thermo Fisher Scientific, USA), following the manufacturer’s instructions. Total RNA concentration was determined by spectrophotometric measurement at 260 nm. Reverse transcription was then performed using Applied Biosystems High-Capacity cDNA Reverse Transcription Kits (Applied Biosystems, USA). Target genes (arginase-1, TNF-α, IL-10) were quantified by SYBR Green qPCR in 20-µL reactions under standard cycling conditions (95 °C denaturation, 60 °C annealing). Expression levels were normalized to β-actin and analyzed via the 2 − ΔΔCT method (PE Biosystems software v1.7, USA) [27]. Primer sequences are provided in Table 1.

**Table 1:** Primer sequences of the studied genes

	Forward primer sequence	Reverse primer sequence
**Arginase 1**	5’-CTTGCGAGACGTAGACCCTG-3’	5’-TCCATCACCTTGCCAATCCC-3’
**TNF-α**	5’-GACCCCTTTACTCTGACCCC-3’	5’-AGGCTCCAGTGAATTCGGAA-3’
**IL-10**	5’-TGAGCAACTATTCCAAACCAGC-3’	5’-CGCAGCTCTAGGAGCATGTG-3’
**β-actin**	5’-CATTGCTGACAGGATGCAGA-3’	5’-CTGCTGGAAGGTGGACAGTGA-3’

### Statistical analysis of collected data

Statistical analysis was performed using Jamovi (version 2.6.44) software. Data of the various study parameters were tested for normality using the Shapiro-Wilk test. Normally distributed data were tested using one way-ANOVA, and comparison between groups was done using Tukey post hoc comparison tests. Non-normally distributed data were analyzed using the Kruskal-Wallis test followed by Dwass-Steel-Critchlow-Fligner (DSCF). Significance was calculated at a P-value of < 0.05.

Since published data on the effect of IVM-NP and MOL-NP and their combination are not satisfactorily available, we have determined the sample size based on our previous observation that free IVM achieved an 84% reduction in *Trichinella spiralis* worm burden compared to controls [20]. Power analysis (α = 0.05, power = 0.8, *d* = 2.0) indicated that 6 mice per group were required.

## Results

### Nanoparticle characterization

#### Ivermectin NPs

**Fig. 1:**
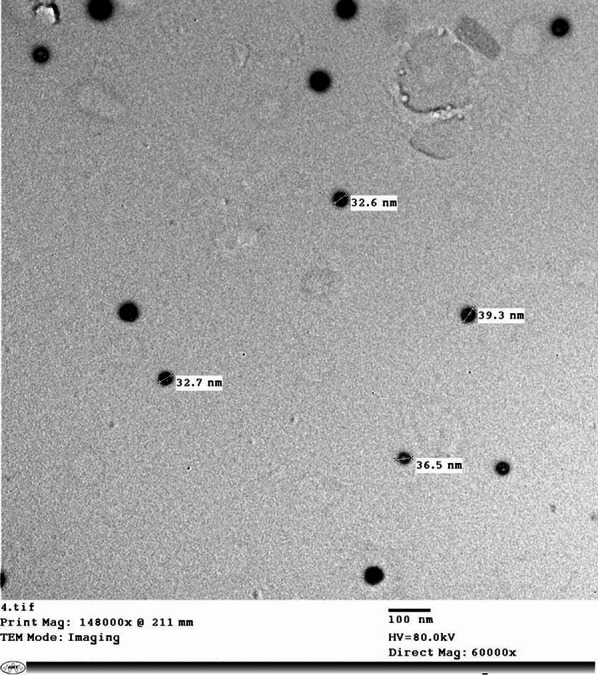
Transmission electron microscopic (TEM) picture of IVM nanoparticles

The particles were uncontrolled in shape with a size range of approximately (32 to 39 nm) with crystal structure suspension and 98.5% purity (Fig. 1).

#### Moringa oleifera nanoparticles

**Fig. 2:**
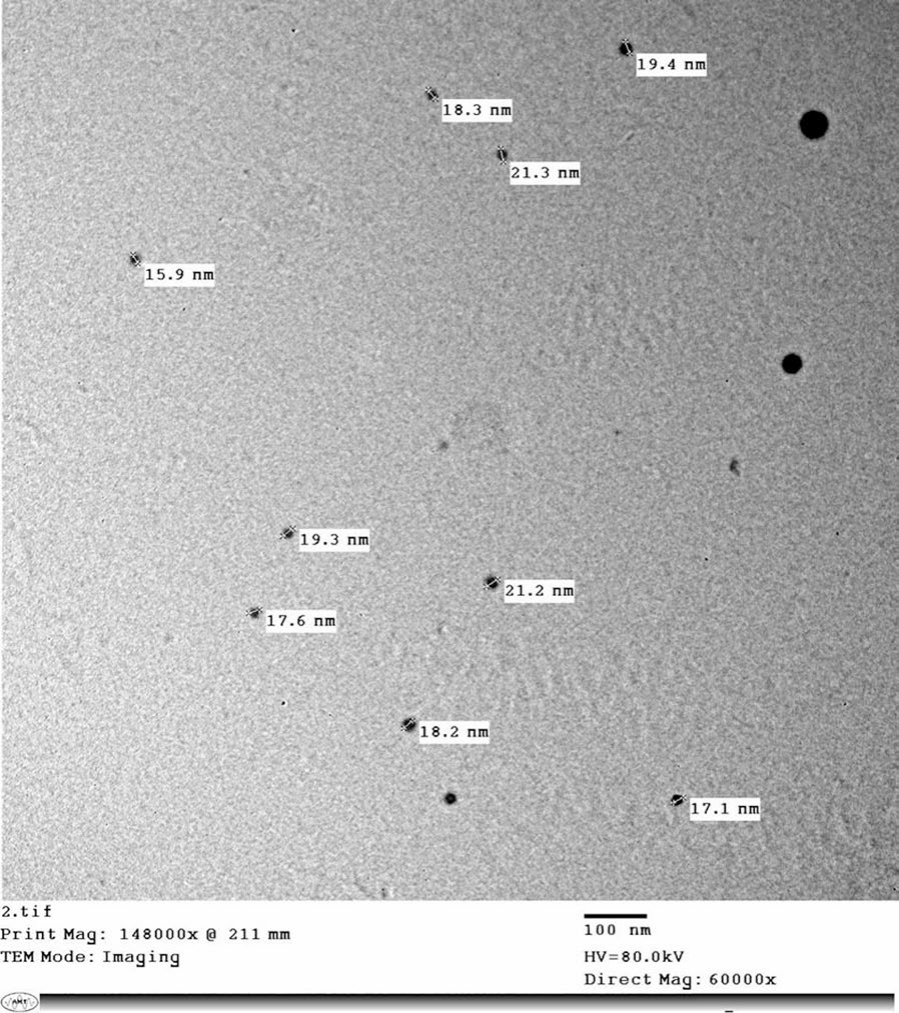
Transmission electron microscopic (TEM) picture of MOL nanoparticles

The particles were uncontrolled in shape with a size range of approximately (15 to 21 nm) with crystal structure suspension and 98.5% purity (Fig. 2). Analysis of MOL components revealed a variety of components represented in Table 2.

**Table 2:** Biochemical contents of MOL

Components	Value (mg/g DW)
Total phenols	1.635
Total chlorophyll	4.378
Ascorbic acid(mg/g–1FW)	8.47
Total flavonoids content (mg/100 g)	0.37
Antioxidant activity (µg/mL)	15.22
Tannins	2.96
Total carotenoids	1.72
Amino acids	387.72
Proline	33.65
Total Omega-6 fatty acids	7.46
Total Omega-3 fatty acids	38.19
Total carbohydrate (g/100 g D.W)	41.5

Nutrient profile	Value (mg/g DW)
Potassium	13.78
Phosphorus	3.82
Nitrogen	12.36
Calcium	15.92
Sodium	54.3
Magnesium	3.96
Zinc	0.051
Iron	0.379
Manganese	0.081
Selenium	0.36
Boron	0.047
Copper	0.038

Phytohormonal profile	Value (µg/g–1FW)
Gibberellins	0.65
Cytokinins	0.63
Indol acetic acid	0.72
Abscisic acid	0.13
Salicylic acid	1.87

### Effect of therapeutic agents on body weight of study animals

**Fig. 3:**
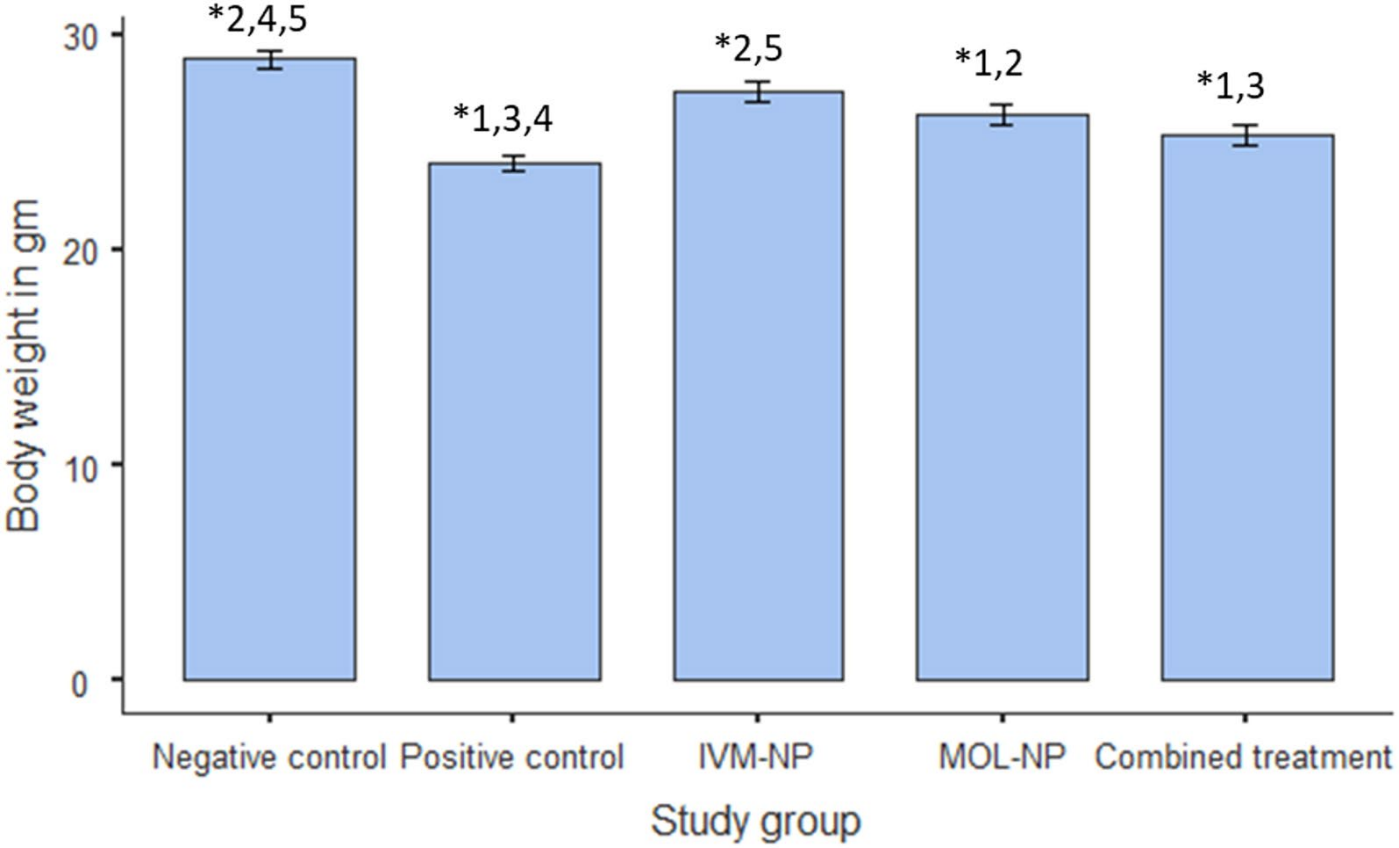
Mean values of mice body weight (gm) in the different study groups. (One-way ANOVA followed by Tukey post hoc test; *Statistical significance at *P* < 0.05. Study groups: 1 = Negative control; 2 = Positive control; 3 = IVM-NP; 4 = MOL-NP; 5 = Combined treatment)

A significant reduction in body weight was observed in *T. spiralis*-infected mice as compared to non-infected mice (*P* < 0.001). Among the treated groups, improvement in weight loss was observed in all treated groups most notably in mice receiving IVM-NP monotherapy (Fig. 3).

### Assessment of parasite burden (adult worm count)

Combined IVM-NP and MOL-NP regimen showed the most prominent reduction in intestinal worm count as compared to the positive control group (80.7% reduction; *P* < 0.05). Despite the significant decrease in worm burden in MOL-NP-treated mice, the percent reduction in worm count was lower than that observed in mice receiving IVM-NP monotherapy or those receiving the combined treatment (*P* < 0.05) (Table 3).

**Table 3:** Adult worm count in intestinal fluid/mouse (Mean ± SD) in infected treated and non-treated groups

Adult worm count in intestinal fluid/mouse				
Group number	Study group	Mean (number of worms/animal)	SD	Reduction in comparison to the positive control group (%)
2	Positive control	95.714^*3,4,5^	5.736	0
3	IVM-NP	23.286^*2,3^	2.870	76.7
4	MOL-NP	56.286^*2,3,5^	5.251	41.2
5	Combined therapy	18.429^*2,4^	10.907	80.7

Kruskal Wallis followed by Dunn’s test with Bonferroni adjustment. * Denotes statistical significance at P value < 0.05 as compared to the corresponding value in the indicated groups: ^2^ = Positive control, ^3^ = IVM-NP, ^4^ = MOL-NP, ^5^ = Combined therapy

### Histopathology of small intestine

**Fig. 4:**
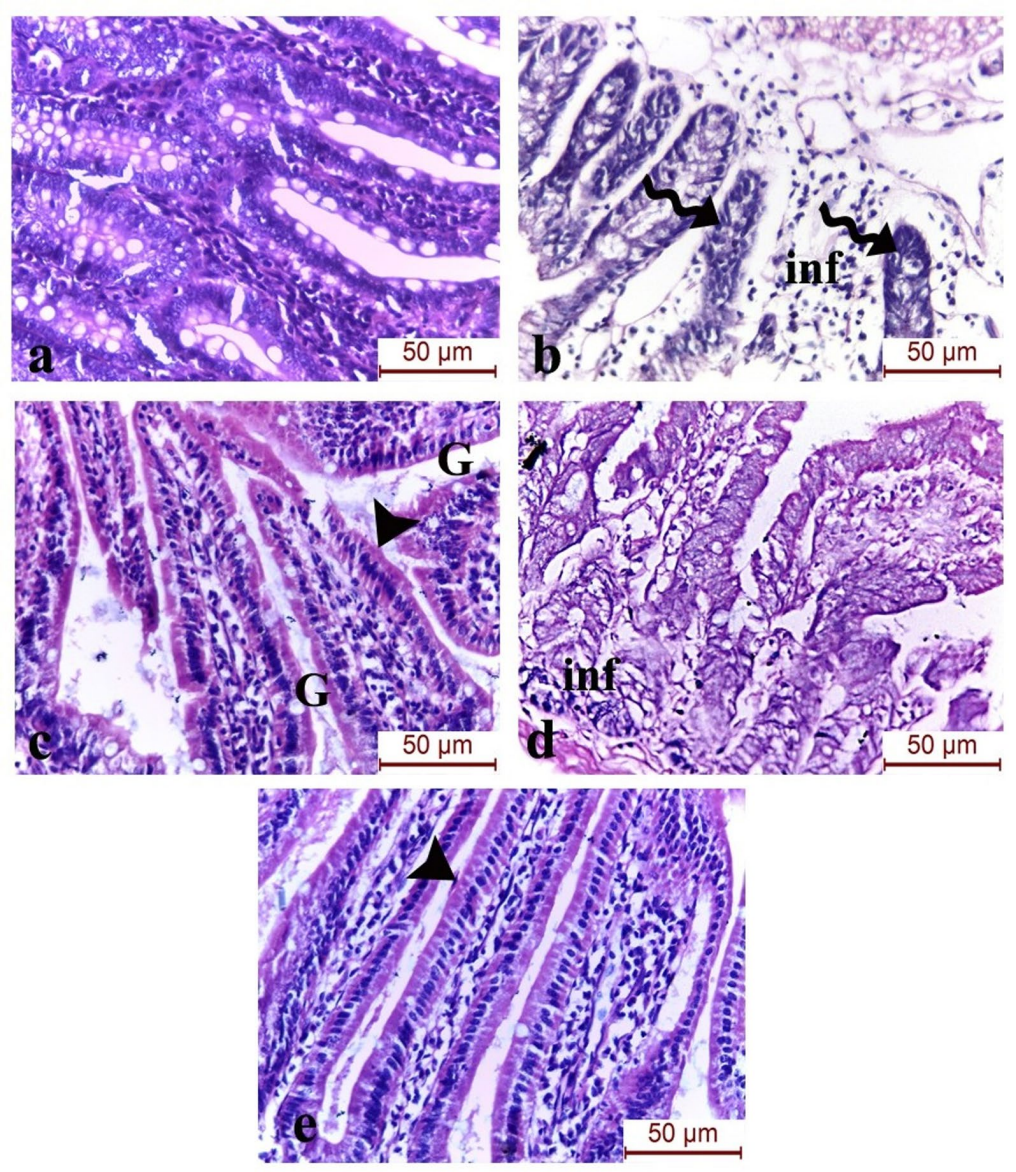
H&E-stained sections in the small intestines of the different study groups stained with H&E (×400). (**a**) Control group, (**b**) *Trichinella spiralis*-infected group showed marked villous atrophy (spiral arrow) with degeneration of the brush border and dense cellular inflammatory cellular infiltration (inf). (**c**) IVP-NP-treated group displayed intestinal structural improvement with regeneration of the brush border (arrow head) and goblet cells (G). (**d**) MOL-NP-treated group showed reduction in the inflammatory infiltrates (inf) with broadening of the villi. (**e**) Combined IVP-NP and MOL-NP-treated group exhibited marked improvement of the architecture of the intestinal villi with brush border restoration (arrow head) (scale bar 50 μm)

Examination of the *Trichinella spiralis*-infected group showed marked attenuation of the intestinal villi with degeneration of the brush border and dense cellular inflammatory cellular infiltration invading the atrophic villi. The IVP-NP-treated group displayed improvement of the intestinal architecture with regeneration of the brush border and goblet cells. The MOL-NP-treated group shows a reduction in the inflammatory infiltrates with broadening of the villi and the combined IVP-NP and MOL-NP-group showed marked improvement of the intestinal villi with brush border restoration (Fig. 4).

### Detection of iNOS and NF-κB protein expression by IHC

**Fig. 5:**
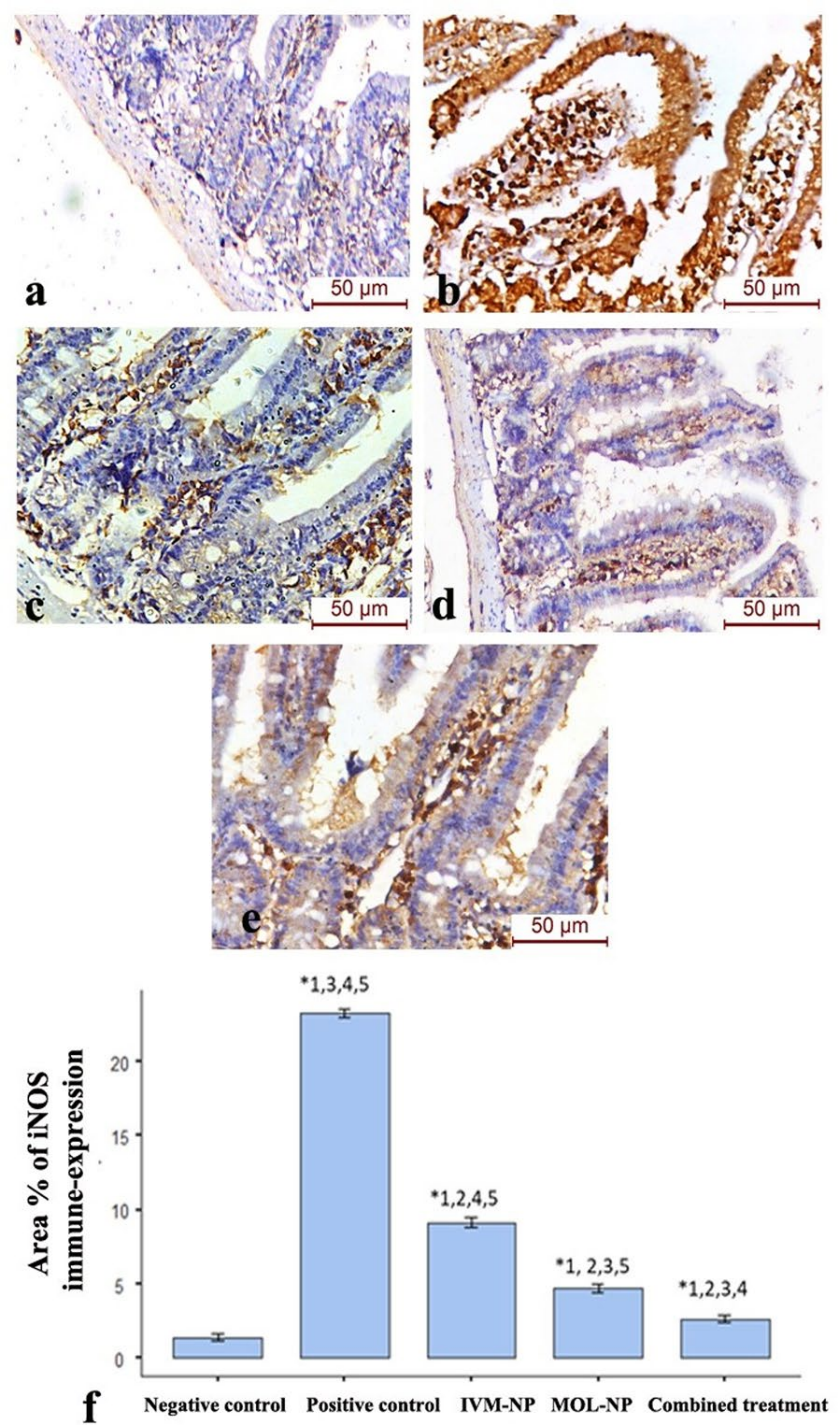
Inducible NOS immunohistochemistry in the intestinal phase of (**a**) Negative control group, (**b**) Positive control (*T. spiralis*-infected) group, (**c**) IVM-NP-treated group, (**d**) MOL-NP-treated group (MOL-NP), (**e**) Combined IVM-NP and MOL-NP-treated group, (**f**) Area percentage of iNOS immune-expression in the different study groups (Significant versus. 1: Negative control; 2: Positive control; 3: IVM-NP; 4: MOL-NP; 5: Combined treatment at *P* < 0.05 using ANOVA followed by Tukey post-hoc test). Strong positive iNOS immune-reactivity was observed in the intestinal mucosa and inflammatory cells of the *T. spiralis*-infected group. Moderate immune-expression was noticed in the lamina propria of the IVM-NP-treated group. Weak immunoreactivity is observed in the MOL-NP and combined therapy group

The expression level of iNOS was significantly increased (p-value < 0.001) in the *Trichinella spiralis*-infected group compared to the control group. The results showed significant decrease in the area percentage of iNOS immunoreactivity in all treated groups compared to the infected group (p-value < 0.001). The maximum inhibition of iNOS immune reaction was observed in the combined IVP-NP and MOL-NP group which was significantly lower as compared to the IVP-NP or MOL-NP monotherapy groups (p-value < 0.01) (Fig. 5).

**Fig. 6:**
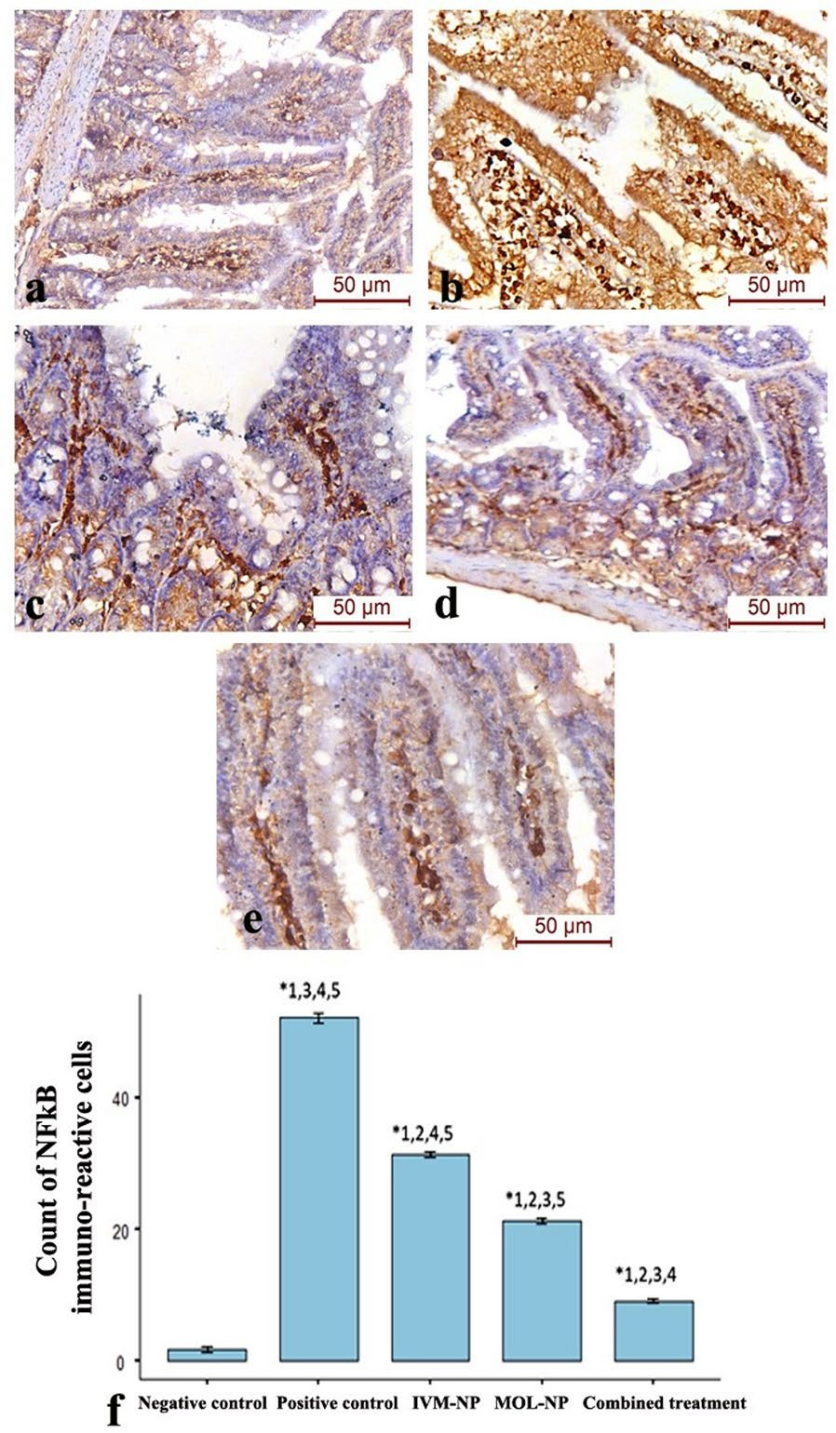
NF-κB immunohistochemistry in the intestinal phase of (**a**) Negative control group, (**b**) Positive control (*T. spiralis*-infected) group, (**c**) IVM-NP-treated group, (**d**) MOL-NP-treated group (MOL-NP), (**e**) Combined IVM-NP and MOL-NP-treated group, (**f**) Area percentage of NF-κB immune-expression in the different study groups (Significant versus. 1: Negative control; 2: Positive control; 3: IVM-NP; 4: MOL-NP; 5: Combined treatment at *P* < 0.05 using ANOVA followed by Tukey post hoc test). Numerous NF-κB immune-reactive cells were observed in the positive control group. Reduction in the NF-κB positive cells was noticed in the IVM-NP, MOL-NP and combined therapy groups

Comparable results have been obtained from the NF-κB immunohistochemical expression, where the count of NF-κB immune-reactive cells in the lamina propria was significantly decreased in the IVP-NP and MOL-NP groups with the maximum reduction observed in the combined therapy group (p-value < 0.001) (Fig. 6).

### Assessment of relative gene expression levels of arginase-1, TNF-α and IL-10

**Fig. 7:**
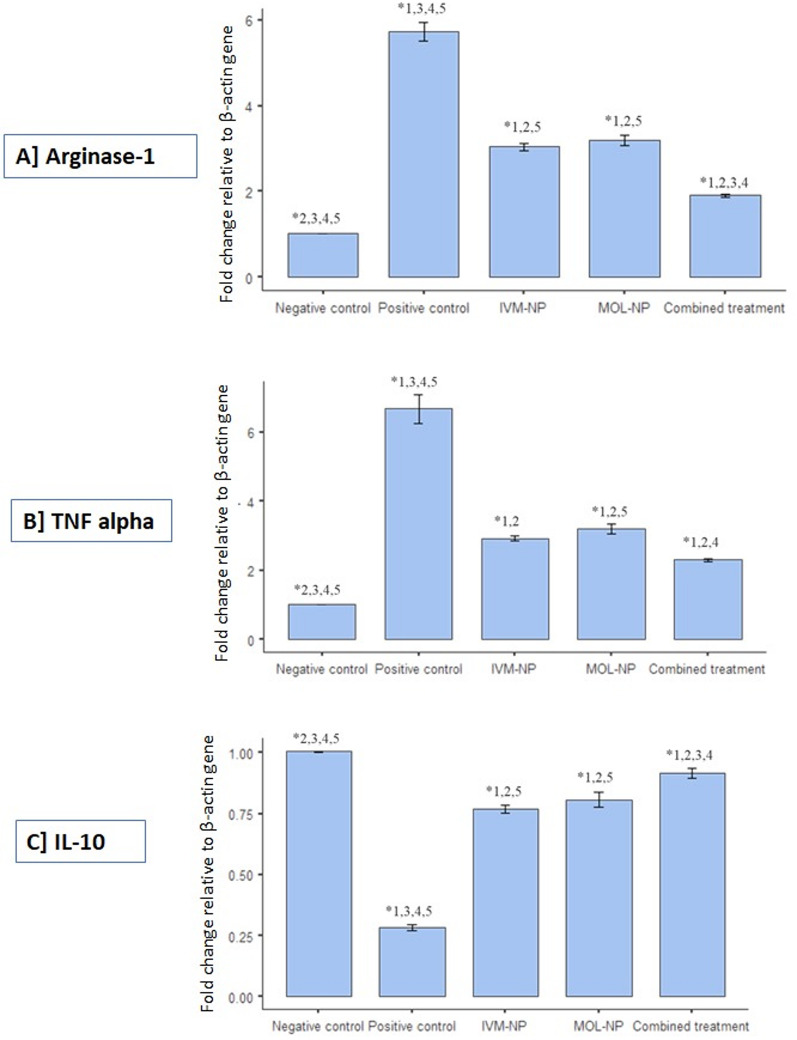
Fold change (Relative gene expression) of (**A**) Arginase-1, (**B**) TNF-α and (**C**) IL-10 in the different study groups. (*Statistical significance at *P* < 0.05 by Kruskal-Wallis followed by pairwise DSCF test; Study groups: 1 = Negative control; 2 = Positive control; 3 = IVM-NP; 4 = MOL-NP; 5 = Combined treatment)

Infection with *T. spiralis* significantly increased the expression of the arginase-1 gene as compared to uninfected mice (*P* < 0.05). While both IVM-NP and MOL-NP significantly reduced arginase-1 expression levels, the most effective reduction in gene expression was observed in the combined therapy groups (*P* < 0.05). No significant difference was observed between the mean values of the relative gene expression levels of arginase-1 in the IVM-NP and MOL-NP groups (*P* = 0.738) (Fig. 7A).

The expression of the TNF-α gene was significantly induced in response to *T. spiralis* infection (*P* < 0.05). A significant reduction of gene expression was observed in all treated groups (*P* < 0.05). No significant difference was observed between gene expression levels in the IVM-NP and MOL-NP groups (*P* = 0.532) (Fig. 7B).

*T. spiralis*-infected mice showed significantly decreased expression levels of the IL-10 gene as compared to uninfected mice (*P* < 0.05). All treated groups showed significantly higher IL-10 levels as compared to the positive control group (*P* < 0.05). However, no significant difference was observed between the mean values of the relative gene expression levels of IL-10 in the IVM-VP and MOL groups (*P* = 0.738). Moreover, no significant difference was observed between the mean relative gene expression values of the MOL-NP and the combined treatment groups (*P* = 0.247) (Fig. 7C).

### Correlation between study parameters

**Fig. 8:**
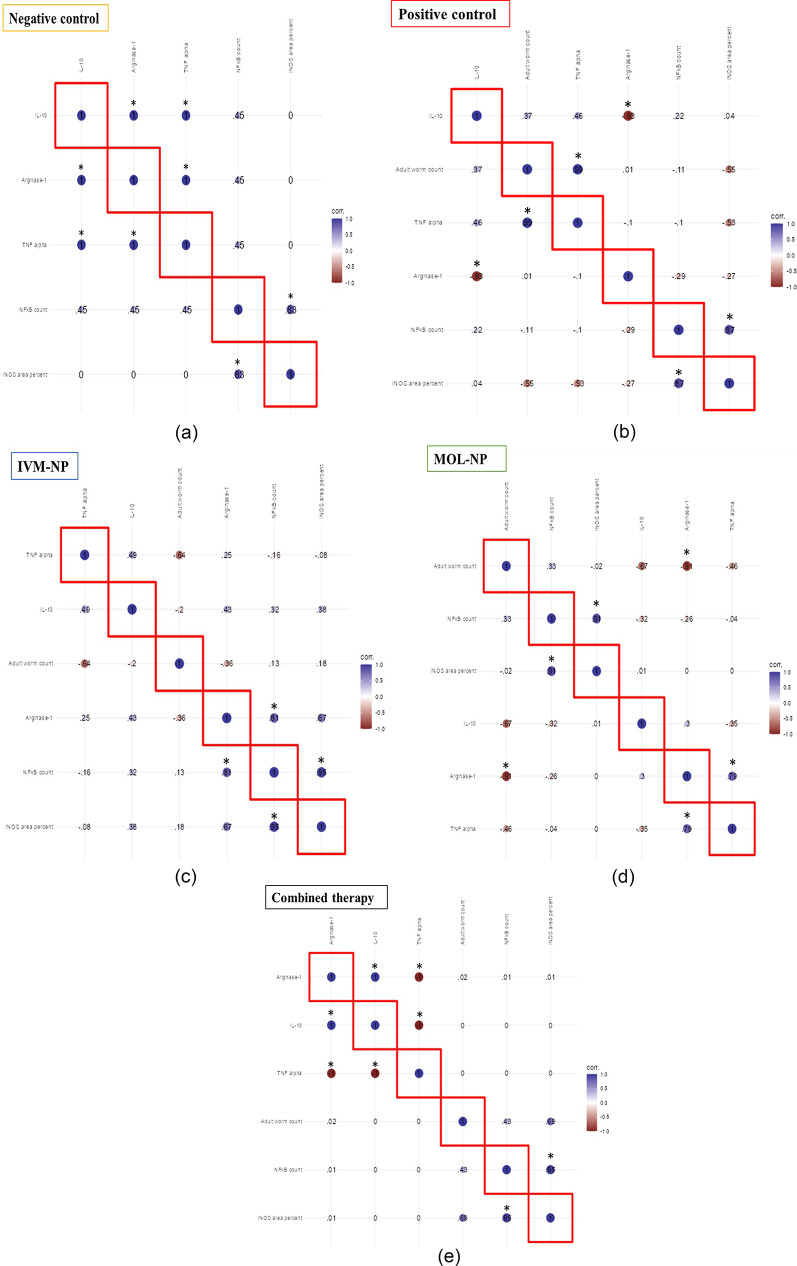
a. Correlation heatmap showing correlation between study parameters in the negative control group using Pearson r correlation test (statistical significance at P<0.05). **b**. Correlation heatmap showing correlation between study parameters in the positive control group using Pearson r correlation test (statistical significance at P<0.05). **c**. Correlation heatmap showing correlation between study parameters in the IVM-NP-treated group using Pearson r correlation test (statistical significance at P<0.05). **d**. Correlation heatmap showing correlation between study parameters in the MOL-NP-treated group using Pearson r correlation test (statistical significance at P<0.05). **e**. Correlation matrix showing correlation between study parameters in the combined IVM-NP and MOL-NP treatment group using Pearson r correlation test (statistical significance at P<0.05)

Figures 8 represent the correlation heatmaps in the different study groups using Pearson r correlation test.

In the negative group, a positive correlation was observed between arginase-1, IL-10 and TNF-α. A positive correlation was also observed between NF-κB and iNOS area percent (*P* < 0.05) (Fig. 8a).

In the positive control group, a significantly negative correlation was observed between IL-10 and arginase-1. A significantly positive correlation was observed between adult worm count and TNF-α levels. The correlation between NF-κB count and iNOS expression was also significantly positive (*P* < 0.05) (Fig. 8b).

In mice receiving IVM-NP, a significantly positive correlation was observed between arginase-1 expression levels and NF-κB count. Again, NF-κB and iNOS area percent were positively correlated (*P* < 0.05) (Fig. 8c).

In mice receiving MOL-NP, a significantly negative correlation was observed between arginase-1 expression levels and adult worm count. The significant positive correlation between NF-κB and iNOS was also observed in this group (*P* < 0.05) (Fig. 8d).

In mice receiving both IVM-NP and MOL-NP, a significantly negative correlation between TNF-α and arginase-1was observed. TNF-α was also negatively correlated with IL-10. A positive correlation was found between IL-10 and arginase-1 gene expression and also between NF-κB and iNOS area pecentage (*P* < 0.05) (Fig. 8e).

## Discussion

Parasitic infections modulate the host immunity by various mechanisms, including the regulation of macrophage polarization and phenotype switching [2]. Owing to the diversity of tissue involvement in *T.spiralis* infection, both M1 and M2 phenotypes contribute to the anti-*Trichinella* immune response as demonstrated by the study conducted by Sun et al. [5]. They conducted a time-dependent study involving different tissues to assess the activity of macrophages using flow cytometry. The authors found that early during infection (between day 1–5 post-infection), the M1 macrophage response was dominating in the small intestine of the study animals. After 15 days of infection, the small intestine showed a mixed M1 and M2 response which was replaced by a predominantly M2 type response during late stages of infection (after 30 days). In our study, protein expression of iNOS, a type 1 macrophage marker, and relative gene expression of arginase-1, a type 2 macrophage marker, were both elevated after 7 days of *T. spiralis* infection, indicating the establishment of a mixed macrophage response during the intestinal phase of the disease. Kang et al. [28] studied the effect of *T. spiralis* on macrophage function and the potential anti-inflammatory effect of parasite-derive excretory-secretory (ES) products on experimentally induced colitis. They found that the peritoneal transfer of macrophages from *T. spiralis* infected animals led to an increased secretion of IL-10 and a suppression of pro-inflammatory cytokine production. In addition, treatment of lipopolysaccharide- induced macrophages with *T. spiralis*-ES products led to the suppression of iNOS production and a shift towards a M2 phenotype.

The role of *T. spiralis* derived proteins in macrophage polarization was also investigated by Liu et al. [29], who investigated the effect of co-incubation of *T.spiralis* cathepsin L with murine macrophages. The authors observed an increased release of iNOS, IL-6, IL-1β and TNF-α, indicating a type 1 macrophage response. Furthermore, the study revealed upregulated expression of phosphorylated NF-κB and its inhibitor IκBα, along with enhanced nuclear translocation of the NF-κB p65 subunit, suggesting activation of the NF-κB signaling pathway. The addition of a NF-κB inhibitor interfered with the macrophage potential to polarize towards a type 1 response.

Management of *T. spiralis* is challenged by the low bioavailability of anti-parasitic drugs, where intestinal inflammation per se decreases the absorption of anti-parasitic agents [30]. Nanotechnology has emerged as a potential solution for improving drug delivery, bioavailability and decreasing drug toxicity [31]. The current study investigated the effect of IVM-NP and MOL-NP on macrophage-related immune activity during intestinal trichinosis. Infection with *T. spiralis* led to a significant increase in arginase-1 and TNF-α gene expression, whereas the relative expression of IL-10 significantly decreased. Both IVM-NP and MOL-NP reversed these effects, and the combination of both therapeutic agents was most effective in decreasing arginase-1 and TNF-α relative gene expression and decreasing the expression of IL-10. The combined treatment was also the most effective in decreasing intestinal worm count. In contrast, dual administration of IVM-NP and MOL-NP were less efficient than the monotherapy of both drugs in attenuating the increased protein expression of NF-κB and iNOS observed after infection with *T. spiralis*.

Nanotechnology has provided a breakthrough in the improvement of anti-parasitic drug delivery. Gamboa et al. [11] demonstrated that IVM lipid loaded nanoparticles achieve a higher peak plasma concentration and longer elimination half-life in the liver, small intestine and lungs when compared to commercial IVM preparations.

Elmehy and his colleagues [32] investigated the effect of 2 different IVM nano-formulations on different stages of *T. spiralis* infection. They employed a niosomal IVM preparation, which is lipid based, and a nano-crystalline IVM formulation. Niosomal IVM was superior in alleviating intestinal inflammation and improving oxidative status during the intestinal phase infection. In addition, it was more efficient than the nano-crystalline preparation in reducing inflammation and angiogenesis, where niosomal IVM achieved a higher reduction in the expression vascular endothelial growth factor (VEGF), matrix-metalloproteinase-9 (MMP-9) and regulated on activation, normal T cell expressed and secreted (RANTES). VEGF is an angiogenesis marker, MMP-9 is a marker of inflammation and tissue remodeling, while RANTES is an inflammatory chemokine that attracts immune cells [32].

The synergistic effect of IVM nanoparticles to conventional antiparasitic therapy was investigated by Moawad et al. [33] by adding IVM chitosan loaded nanoparticles to albendazole in the treatment of *T. spiralis*-infected mice. They observed that the combination of both agents was most efficient in decreasing the intestinal worm count and the expression of IFN γ, TNF-α and iNOS genes. Similar to our study, an increase in IL-10 expression was also observed.

Faheem et al. [34] investigated the effect of MOL and MOL loaded on chitosan NP in comparison to metronidazole therapy on parasite burden and intestinal pathology in experimental giardiasis. Although MOL significantly decreased the *G. lamblia* cyst count (76.89% reduction), it was not as effective as metronidazole therapy (79.76% reduction). MOL NPs, however, were superior to both drug regimen in reducing parasite count (86.01%). Nevertheless, MOL both alone and as NP preparation was more efficient in improving intestinal pathology than metronidazole.

Abdel-Latif et al. [35] demonstrated that *Moringa oleifera* extracts (MOE) had an immunomodulatory and protective effect against intestinal *Hymenolepis nana* infection by exerting an anti-oxidant response and inducing T helper 2 cytokine expression. Mice immunized with MOE prior to infection showed attenuated intestinal pathology, increased goblet cells, mast cells and secretory IgA, in addition to decreased parasite shedding.

The biological actions of *Moringa oleifera* are attributed to the wide array of its biochemical constituents, such as flavonoids, phenols, polysaccharides, amino acids, minerals and phytohormones. Flavonoids were found to exert an immune-modulatory effect during experimental *T. spiralis* infection by decreasing the intestinal expression of NOD-like receptor-pyrin domain containing 3 (NLRP3). This intracellular protein is critical for the inflammasome formation as part of the innate immune response and is dependent on NF-κB for its upregulation [36]. Flavonoids were also investigated as potential anti-cancer agents, due to their effects on angiogenesis, cell signaling and cell death [37].

Polyphenols present in *Moringa oleifera* contribute to the antioxidant potential of the plant. In addition to flavonoids and phenols, anti-inflammatory and anti-bacterial effects are also exerted by tannins, saponins and vitamins, particularly vitamin C [38]. MOL polysaccharides were found to have a regenerative effect on intestinal immunopathological changes as represented in an experimental colitis model studied by Hussein et al. [39]. MOL polysaccharides significantly improved the pathological changes resulting from dextran sulfate induced colitis in mice. In addition, induction of the mRNA expression of the anti-inflammatory cytokine IL-10 and inhibition of the mRNA expression of pro-inflammatory cytokines such as TNFα and IL-1β was observed. Moreover, the authors demonstrated that the anti-inflammatory effect of MOL was also achieved by inhibiting the TLR4/MyD88/NF-κB signaling pathways.

The incorporation of herbal medicines in the management of parasitic infections has re-surfaced to overcome limitations of conventional anti-parasitic agents, such as low bioavailability, toxicity and development of resistance [40]. Literature on the combination of IVM and *Moringa oleifera* is relatively scarce, especially regarding their nanoparticle preparations. Uko et al. reported that MOL extracts and vitamin C improved the reproductive toxicity induced by IVM in rabbits through their antioxidant properties [41]. Similarly, Boudjema et al. stated that MOL extracts improve the oxidative damage produced by abamectin on the brains and erythrocytes of rats [42].

## Conclusion, limitations and recommendations

In conclusion, this study illustrated a pivotal role of IVM-NP and MOL-NP in immune regulation by significantly downregulating pro-inflammatory markers (iNOS and TNF-α) while upregulating the anti-inflammatory cytokine IL-10 in *T.spiralis* infection. Furthermore, combination therapy synergistically decreased parasite burden and intestinal pathology more effectively than monotherapies. While this study demonstrates promising anti-parasitic effects of both nanoparticle preparations and their combination, future studies targeting the effect of batch variability and nanoparticle properties on the desired drug effects are recommended. The notable effects of IVM-NP on the adult worm burden and inflammatory response during the intestinal phase, strongly encourages the design of additional studies on the effect of this agent on the muscle phase of the disease, posing the question on whether the employment of nanotechnology can aid to overcome the relatively limited efficacy of IVM on encysted *T. spiralis* larvae.

## Data Availability

No datasets were generated or analysed during the current study.
